# Detection of cancer antigens (CA-125) using gold nano particles on interdigitated electrode-based microfluidic biosensor

**DOI:** 10.1186/s40580-019-0173-6

**Published:** 2019-01-17

**Authors:** Bharath Babu Nunna, Debdyuti Mandal, Joo Un Lee, Harsimranjit Singh, Shiqiang Zhuang, Durgamadhab Misra, Md Nasir Uddin Bhuyian, Eon Soo Lee

**Affiliations:** 1 Advanced Energy Systems and Microdevices Laboratory, Department of Mechanical and Industrial Engineering, New Jersey Institute of Technology, 200 Central Avenue, Rm MEC 327, Newark, NJ 07102-1982 USA; 2 Provost Summer Research Intern at New Jersey Institute of Technology & Tenafly High School, Tenafly, NJ USA; 3 Department of Electrical and Computer Engineering, New Jersey Institute of Technology, Newark, NJ 07102 USA

**Keywords:** Biosensor, Interdigitated gold electrodes, Microfluidic channel, Gold nanoparticles, Capacitance measurements

## Abstract

Integrating microfluidics with biosensors is of great research interest with the increasing trend of lab-on-the chip and point-of-care devices. Though there have been numerous studies performed relating microfluidics to the biosensing mechanisms, the study of the sensitivity variation due to microfluidic flow is very much limited. In this paper, the sensitivity of interdigitated electrodes was evaluated at the static drop condition and the microfluidic flow condition. In addition, this study demonstrates the use of gold nanoparticles to enhance the sensor signal response and provides experimental results of the capacitance difference during cancer antigen-125 (CA-125) antigen–antibody conjugation at multiple concentrations of CA-125 antigens. The experimental results also provide evidence of disease-specific detection of CA-125 antigen at multiple concentrations with the increase in capacitive signal response proportional to the concentration of the CA-125 antigens. The capacitive signal response of antigen–antibody conjugation on interdigitate electrodes has been enhanced by approximately 2.8 times (from 260.80 to 736.33 pF at 20 kHz frequency) in static drop condition and approximately 2.5 times (from 205.85 to 518.48 pF at 20 kHz frequency) in microfluidic flow condition with gold nanoparticle-coating. The capacitive signal response is observed to decrease at microfluidic flow condition at both plain interdigitated electrodes (from 260.80 to 205.85 pF at 20 kHz frequency) and gold nano particle coated interdigitated electrodes (from 736.33 to 518.48 pF at 20 kHz frequency), due to the strong shear effect compared to static drop condition. However, the microfluidic channel in the biosensor has the potential to increase the signal to noise ratio due to plasma separation from the whole blood and lead to the increase concentration of the biomarkers in the blood volume for sensing.

## Introduction

An electrical biosensor detects biomolecular reactions by measuring changes in electrical properties like voltage, current, impedance, capacitance etc., [1]. Measuring capacitance has advantages such as high sensitivity to small changes in dielectric parameters, the possibility of minimizing the sensor size, and low power consumption requirement [2]. The integration of microfluidics and electrical immuno biosensing has growing demand due to its potential to reduce processing time and have low reagent consumption [3]. Bange et al. [4] was the first group to integrate microfluidics into electrochemical protein immune assays. The immuno biosensing on the microfluidic platform helped to make the electrochemical biosensing assays portable which allows the sensing mechanism to be easily implemented in point of care devices [5]. The implementation of the biosensing in the microchannels significantly reduces the sample requirement from milliliter (ml) to microliter (µl). A low sample volume is highly desired for the bodily fluid samples such as blood. The incorporation of the microfluidic platform on the biosensor provide the feasibility of expanding the sensor to multiplex assay to detect the panel of protein biomarkers that minimize the false positive and false negative scenarios in cancer diagnosis which commonly arise from measuring a single biomarker [6–8]. Though there has been many researchers reported on the integration of the microfluidics to the immunosensors, most of the studies are performed on microfluidic flow driven by the external pumps and flow control devices [9, 10]. In this current study the flow in the microchannel is self-driven and controlled by altering the hydrophilicity of the microchannel. The primary factors contributing to the microfluidic biosensors performance are probe immobilization, specific binding, and the fundamental limits of probe affinity. Due to the tight confinement of the flow of the antigen solution in the microscale, the flows in the microchannel exerts high shear stresses on the surface of the microchannel and influence the stability of the immobilized antibodies on the surface [11, 12]. So the study of the sensing signal response during the flow condition attracted many researchers to develop novel techniques of antibody immobilization for enhanced stability.

For enhanced binding capabilities of the biomolecules on the sensing platform, nanoparticles and nanotechnology have attracted attention in recent years for their potential applications. The recent technology advancement in the microfluidic and nano technology present multiple opportunities for the development of lab-on-chip (LOC) systems to perform a complete set of biomedical assays to achieve the low cost, highly sensitive point-of-care diagnostics [13–16]. Nanoparticles are favorable for biosensing, due to their potential for unique surface chemistry, electrical properties, and being in the same size range as biomolecules. Certain nanoparticles are biocompatible, which enables them to bond with various functional groups like proteins, ligands, peptides, DNA, fatty acids and plasmids for serving the sensing purpose [17, 18]. There are various noble metals like gold, silver, palladium, rhodium, platinum etc. which are biocompatible [19]. Although many noble metals can function as biosensors, gold nanoparticles show promise for biosensing due to their unique surface chemistry, high electron densities, chemical inertness, and their possession of good electrical and optical properties [4, 6–8, 20–48]. The gold nanoparticles help in improving the sensitivity and actively targets the biomarker as it provides the platform for high surface to volume ratio [21, 22]. In label free biosensors, the capacitive measurements are expected to change significantly with different properties such as the dielectric constants [1]. CA-125, a widely used biomarker for detection and monitoring of the ovarian cancer, is an exceptionally large protein (200 to 2000 kDa due to high variable glycosylation) [23, 24]. As the capacitance measurement during the biological interactions is directly influenced by the physiochemical properties of an individual protein, the study of the CA-125 protein detection with its unique properties has gained importance in the research of biosensing.

Daniels et al. [25] has reported that the microfluidic biosensor performance can be enhanced with further research on the probe immobilization techniques. An improved understanding of the relationship between the antibody binding and the capacitance change would enable improved biosensor design and sensitivity. Though there are multiple recent studies performed by researchers, such as Goddard and Erickson [26], who have studied the stability of the biomolecules under the shear flow condition, most of them are limited to the study of DNA immobilization. Though the major targeted probes of the biosensors are proteins and DNA, there is very limited study reported on the antibody immobilization under the shear flow conditions during self-driven flow. This paper reports the sensitivity study of the antigen detection under shear flow condition during self-driven flow of antigen solution, when immobilized with the gold nano particles (GNPs). The sensitivity study performed in this paper on the capacitive signal response of the gold nano particle coated interdigitated electrodes compared to the plain interdigitated electrodes during the antigen–antibody conjugation provide the experimental evidence to the research of nano particle influence on the biosensor research. The study of sensing signal response of antigen–antibody conjugation with multiple concentrations of the antigens discussed in this paper, pave the way for study of sensitivity and specificity of the detection. Also, the change in the sensing signal due to the microfluidic flow of antigen solution when compared to static drop condition during the antigen–antibody conjugation aid the microfluidic biosensing researchers to understand the influence of the shear stress on the biosensing in microchannel.

## Methods/experimental

### Chemicals and apparatus

Thiourea (CH_4_N_2_S), phosphate buffer saline (PBS), 1-ethyl-3-(-3-dimethylaminopropyl) carbodiimide (EDC), *N*-hydroxysuccinimide (NHS), and carboxylic functionalized (Lipoic acid) gold nanospheres were purchased from NanoComposix (USA). The CA-125 monoclonal antibodies and the CA-125 antigens were bought from Meridian Life Science. The polydimethylsiloxane (PDMS) base and curing agent were bought from Fisher Scientific.

### Interdigitated electrodes fabrication

**Fig. 1:**
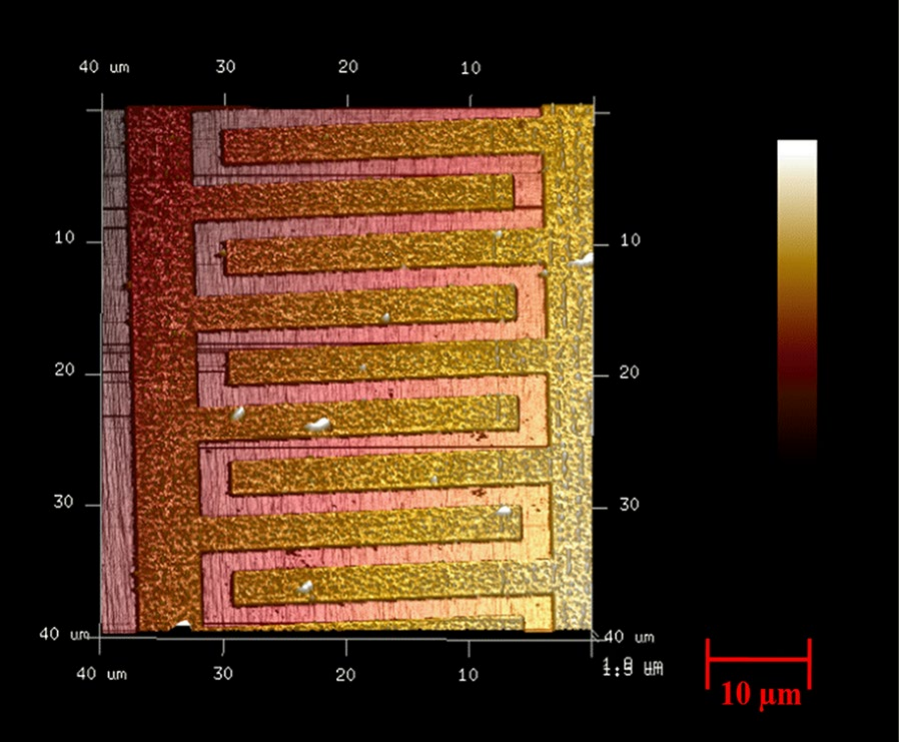
AFM image of the plain electrodes

The interdigitated electrodes were patterned using a photolithography process. A silicon wafer with an oxide layer was used as the substrate. A positive tone photoresist, PMMA-6 was added on top of the silicon wafer and spin coated for 60 s at 3000 rpm. The photoresist coated silicon wafer was exposed with e-beam lithography using JEOL JBX6300-FS Electron Beam Lithography equipment from Brookhaven National Laboratory. After the e-beam exposure, the patterned silicon wafer was developed using 1:3 of MIBK: IPA for 60 s and further washed with IPA for another 60 s followed by drying with nitrogen gas. A layer of Titanium approximately 15 nm thick was deposited on top of the patterned silicon substrate. The purpose of this procedure is to enhance the adhesion of gold on the silicon wafer. Subsequently, a gold layer 95 nm thick was deposited on top of the Titanium coated silicon substrate using high vacuum e-beam metal evaporator (Kurt J. Lesker PVD-75 Evaporator). Followed by the metal depositions, a lift-off process was performed where the sacrificial positive photo resist was removed by cleaning the substrate in an acetone ultrasonic bath for 2 min and then followed by rinsing with IPA and de-ionized water to prevent redeposit ion. Figure 1 shows the AFM image of the plain interdigitated electrodes.

### Insulation of electrodes and addition of gold nanoparticles

**Fig. 2:**
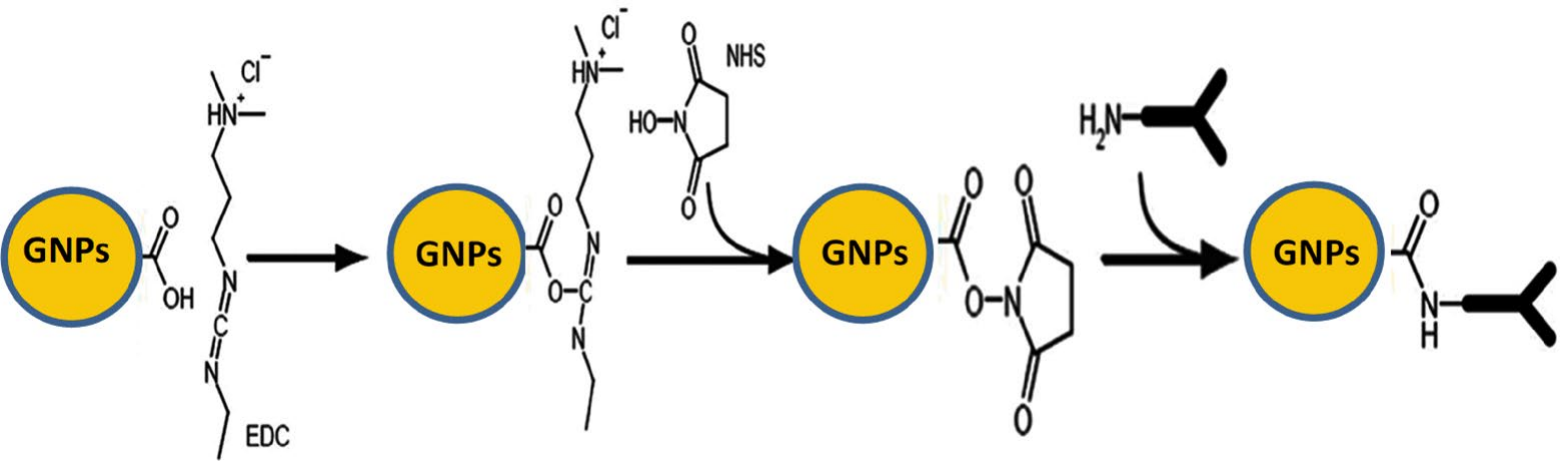
Surface activation process of the carboxylic functionalized gold nanoparticles for antibodies binding

The interdigitated gold electrodes were washed multiple times with ethanol and de-ionized water and dried with nitrogen gas before addition of the self assembled monolayer (SAM) layer. The interdigitated gold electrodes surface was coated with SAM by submerging the sensor in 50 mM thiourea solution overnight (~ 12 h). The interdigitated electrodes were rinsed with ethanol and de-ionized water and dried with nitrogen gas. The formation of the SAM layer was confirmed by taking electrical measurements using a two-point electrical probe station. Furthermore, the AFM image of the electrodes confirmed the formation of the SAM layer. The carboxylic encapsulated gold nanoparticles having 5 nm size were incubated on the surface of the SAM modified sensor for 12 h. After the incubation step, the gold nanoparticles surface was activated using 50 mM of EDC and NHS. This process enables the antibodies to attach covalently to the carboxylic gold nanoparticles [27]. The surface activation of the carboxylic gold nanoparticles is shown in Fig. 2.

### Immobilization of CA-125 antibodies

The sensor surface was washed using PBS solution and dried with nitrogen gas for removal of excess gold nanoparticles. Following this step, the CA-125 antibodies were immobilized on top of the surface activated gold nanoparticles by incubating it with 0.5 µl of 7 mg/ml CA-125 antibodies in PBS solution, for 4 h. The incubation step took place at 4 °C. The sensor surface was rinsed using PBS solution and the non-reacted groups on the sensor surface were blocked by addition of approximately 1 µl of ethanolamine on top of the modified sensor for 1 h. The sensor was further cleaned with PBS and de-ionized water.

### Fabrication of PDMS microchannel

The fabrication of the microchannel is primarily performed in two steps. In the first step, a Si-mold with the required microchannel pattern is fabricated. A positive photoresist (SPR 955) is used to have the photoresist remain only at the microchannel area after UV exposure using the UV mask aligner (since the mask is chromed at the microchannels). The Si-wafer is then etched with deep reactive ion etching (DRIE) to a depth of 107 µm except at the microchannel surface area that is covered with photoresist. By etching the surface unprotected by the photoresist, the negative of the microchannels protrude from the surface of Si-wafer.

The microchannels are fabricated out of polydimethylsiloxane (PDMS) using the Si-wafer mold with negative microchannel. The PDMS base and curing agent are mixed in 10:1 ratio and degassed in the vacuum chamber. The PDMS mixture is poured on the Si-wafer with microchannel and baked for 45 min at 100 °C. The PDMS is then peeled from the Si-wafer and treated with plasma for 100 s to convert the hydrophobic nature of PDMS to hydrophilic. The inlet and outlet ports of the microchannel are holes of 500 μm diameter. The PDMS is then attached to the Si-wafer with the nano circuit to close the channel.

### Addition of CA-125 antigens

**Fig. 3:**
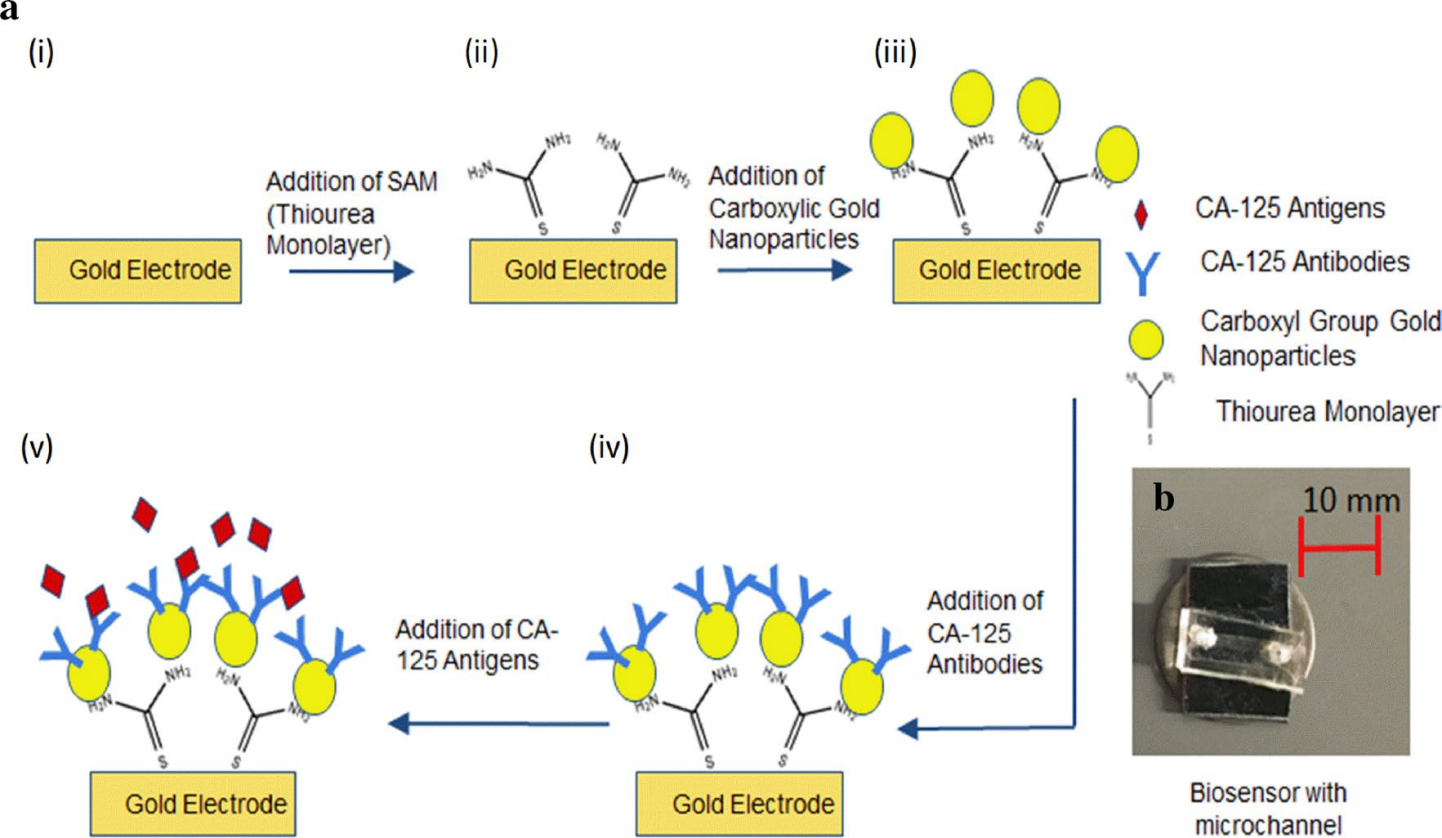
**a** Schematic representation at various stages of biosensor fabrication: (i) Bare electrodes (ii) SAM layer on the bare electrodes (iii) immobilized gold nano particles on the SAM layer (iv) Antibody immobilization on the electrodes (v) antigen–antibody conjugation on the electrodes. **b** Real image of the biosensor with microchannel

The biosensor was exposed to CA-125 antigens in both the static and microfluidic flow conditions. The static condition was achieved by placing a drop directly on the sensor, whereas the flow condition was achieved by placing a drop on the flow inlet, and it followed the channel to encounter the sensor [45–47]. A 5 µl droplet was used in both cases. Figure 3 shows the schematic of the different layers in the biosensor including the SAM layer fabrication, attachment the gold nano particles, immobilization of the CA-125 antibodies and CA-125 antigen–antibody conjugation.

### Electrical measurements

All the electrical measurements in this experiment were taken using a two-point probe station and the capacitance was measured using an Agilent 4284A Precision LCR meter. The selected frequency range was between 10 and 100 kHz with 10 kHz steps. The capacitive values were measured for (a) bare electrodes (b) after insulation of the electrodes by SAM layer, (c) after addition of carboxylic functionalized gold nanoparticles, (d) after the immobilization of the CA-125 antibodies, (e) after the interaction of the PBS solution with the sensor surface with the immobilized CA-125 antibodies, (f) after conjugation of CA-125 antigens and antibodies when antigens solution with different concentration of CA-125 antigens were dropped on the sensing surface, and (g) after conjugation of CA-125 antigens and antibodies when antigens solution flows through the microchannel. All the capacitive measurements were done at 100 mV amplitude with the 0.5 V voltage during this experiment.

## Results and discussion

### Interdigitated electrodes

The interdigitated electrodes produce certain electric fields when voltage is applied, for capacitive measurements. The interdigitated electrodes provide greater effective surface area within the same volume or space which would drastically reduce the sensing setup and cost as compared to other capacitive measurement systems [28–31]. The electric field produced by the interdigitated electrodes is within the nanoscale range, which falls in the region of interest as the size of the antigens and antibodies lies in this range [32, 34]. The dielectric properties of the medium between the interdigitated electrodes provide the electrical information such as conductivity, permittivity, capacitance and impedance. The electric field lines produced by the interdigitated electrodes depend on the electrical input, dielectric medium, and the geometry of the electrodes.

**Fig. 4:**
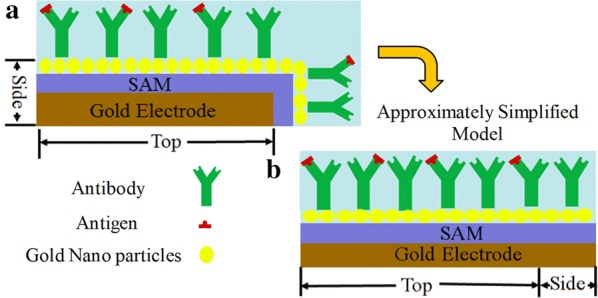
**a** Schematic of the electrode with active ‘Top’ and ‘Side’ surfaces of the and **b** the approximately simplified model of **a** with single surface model for both Top and Side in this analysis

The effective surface area of the electrode that contributes to the capacitance is, ‘Top’ surface area and ‘Side’ surface area as shown in Fig. 4a. The net capacitance ‘C’ of the active surface area (Top + Side) of the electrodes in the comprised capacitance model can be determined by Eq. (1),

1 $${\text{C}} = \upvarepsilon_{{{\text{r}}_{\text{T }} }} \cdot \upvarepsilon {\text{o}} \cdot \left( {\frac{{{\text{A}}_{{{\text{eff}}_{\text{Top}} }} }}{{{\text{d}}_{{{\text{eff}}_{\text{Top}} }} }}} \right) + \upvarepsilon_{{{\text{r}}_{\text{S }} }} \cdot \upvarepsilon {\text{o}} \cdot \left( {\frac{{{\text{A}}_{{{\text{eff}}_{\text{Side}} }} }}{{{\text{d}}_{{{\text{eff}}_{\text{Side}} }} }}} \right)$$

where $\upvarepsilon_{{{\text{r}}_{\text{T }} }}$ and $\upvarepsilon_{{{\text{r}}_{\text{S }} }}$ is the relative permittivity of the material on the top and side surfaces of the electrode, $\upvarepsilon {\text{o}}$ is the vacuum permittivity, ${\text{A}}_{{{\text{eff}}_{\text{Top}} }}$ and ${\text{A}}_{{{\text{eff}}_{\text{Side}} }}$ are the effective surface areas on the top and side surfaces of the electrode, and ${\text{d}}_{{{\text{eff}}_{{_{\text{Top}} }} }}$ is the effective distances neighboring two top surfaces and ${\text{d}}_{{{\text{eff}}_{{_{\text{Side}} }} }}$ is between the adjacent two side surfaces of the electrode.

When the SAM, Antibody and Antigen/Antibody layers are assumed to be homogenous over the surface of electrode, the capacitance of the circuit can be calculated from the equivalent model, with the single surface (Top + Side) as shown in Fig. 4b.

Hence, the net capacitance ‘C’ of the approximately simplified model can be calculated as per Eq. (2),

2 $${\text{C}} = \upvarepsilon_{\text{r}} \cdot \upvarepsilon {\text{o}} \cdot \left( {\frac{{{\text{A}}_{\text{eff}} }}{{{\text{d}}_{\text{eff}} }}} \right)$$

And,

3 $${\text{C}} = {\text{C}}_{\text{Top}} {\text{ + C}}_{\text{Side}}$$

where,

4 $${\text{C}}_{\text{Top}} = \upvarepsilon_{{{\text{r}}_{\text{T }} }} \cdot \upvarepsilon {\text{o}} \cdot \left( {\frac{{{\text{A}}_{\text{Top}} }}{{{\text{d}}_{\text{Top}} }}} \right)$$

5 $${\text{C}}_{\text{Side}} = \upvarepsilon_{{{\text{r}}_{\text{S }} }} \cdot \upvarepsilon {\text{o}} \cdot \left( {\frac{{{\text{A}}_{\text{Side}} }}{{{\text{d}}_{\text{Side}} }}} \right)$$

By assuming,

6 $$\upvarepsilon_{{{\text{r}}_{ \, } }} \approx \upvarepsilon_{{{\text{r}}_{\text{T }} }} \approx \upvarepsilon_{{{\text{r}}_{\text{S }} }}$$

7 $${\text{A}}_{\text{eff}} \approx {\text{A}}_{{{\text{eff}}_{{_{\text{Side}} }} }} + {\text{A}}_{{{\text{eff}}_{{_{\text{Top}} }} }}$$

**Fig. 5:**
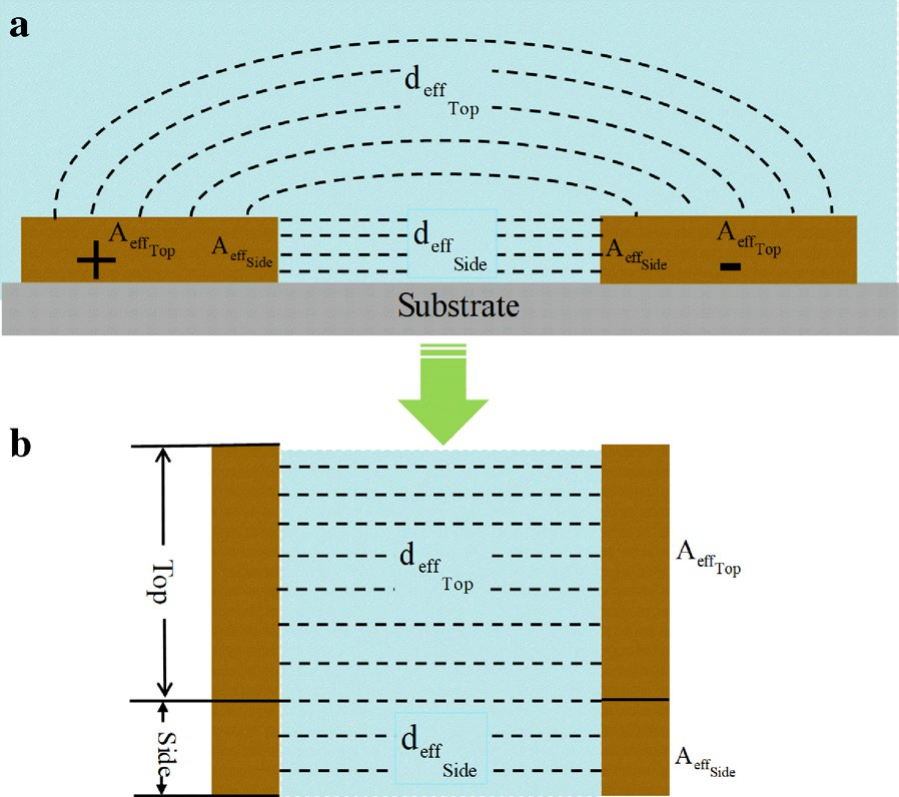
**a** Schematic of the electrode with active ‘Top’ and ‘Side’ surfaces of the and **b** the schematic of the approximately simplified model of **a** with single surface model for both Top and Side in this analysis

With Eq. (2) to Eq. (7) d_eff_ can be written as,

8 $${\text{d}}_{\text{eff}} \approx \frac{{{\text{A}}_{{{\text{eff}}_{{_{\text{Side}} }} }} + {\text{A}}_{{{\text{eff}}_{{_{\text{Top}} }} }} }}{{\left( {\frac{{{\text{A}}_{\text{eff}} }}{{{\text{d}}_{\text{eff}} }}} \right)_{\text{Side}} + \left( {\frac{{{\text{A}}_{\text{eff}} }}{{{\text{d}}_{\text{eff}} }}} \right)_{\text{Top}} }}$$

where, $\upvarepsilon_{{{\text{r}}_{ \, } }}$ (as per Eq. (3)) is the relative permittivity of the material between the electrodes, $\upvarepsilon o$ is the vacuum permittivity, A_eff_ is the effective overall surface area of top and side, and d_eff_ is the effective overall distance between electrodes as shown in Fig. 5 as per Eq. (8).

### Surface characterization of different layers of the biosensor

**Fig. 6:**
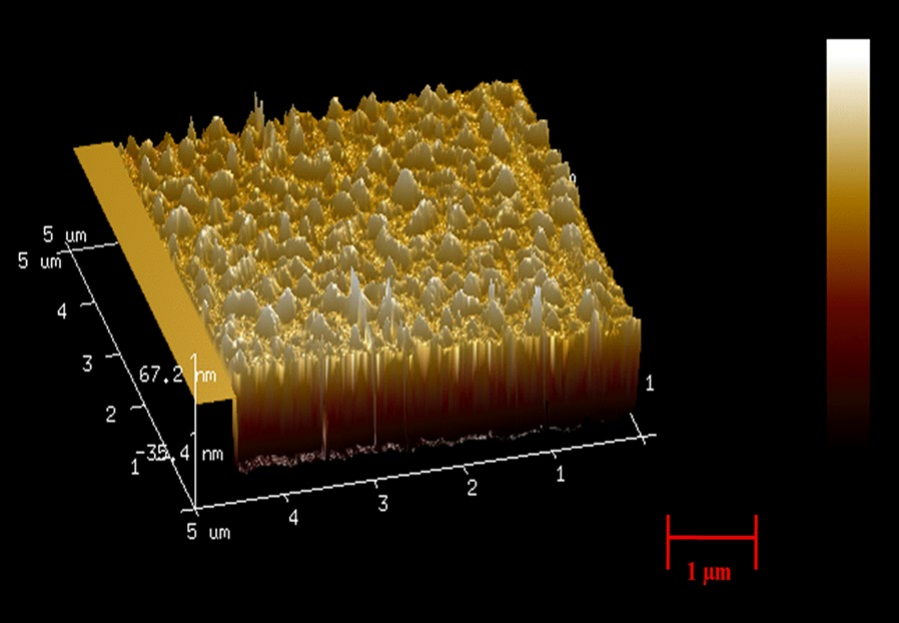
The AFM image of the interdigitated gold electrode coated having SAM layer deposited on top

**Fig. 7:**
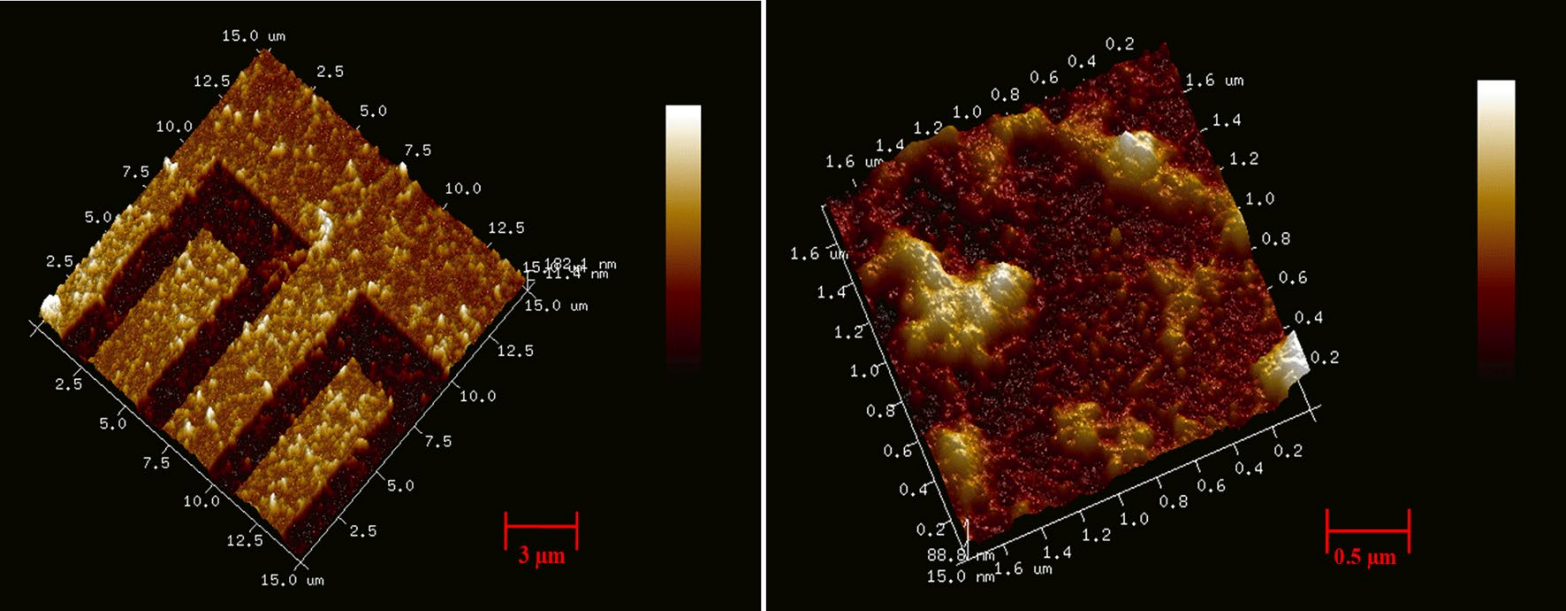
AFM images of the Gold nanoparticles present on top of gold electrodes (Left) and the CA-125 antibodies present on top of the gold nanoparticles in the sensor platform (Right)

In this study, the purpose of the SAM layer is to insulate the electrodes and prevent them from short circuiting [33]. The functionality and the presence of the SAM layer are confirmed by AFM image of the Thiourea coated gold electrodes. Figure 6 shows the AFM image of the electrodes having Thiourea layer deposited on top of it. The increment in the net vertical height (~ 20 nm) of the electrodes along with the surface roughness confirms the formation of the SAM layer. The SAM layer insulation was also confirmed using electrical measurements. The carboxylic functionalized gold nanoparticles were incubated, and surface activated using EDC/NHS coupling. The CA-125 antibodies are then added and incubated on top of the gold nanoparticles. Figure 7 shows the AFM images of the gold nanoparticles and the antibodies present on top of the gold nanoparticles.

### CA-125 antigen solution flow in microchannel

**Fig. 8:**
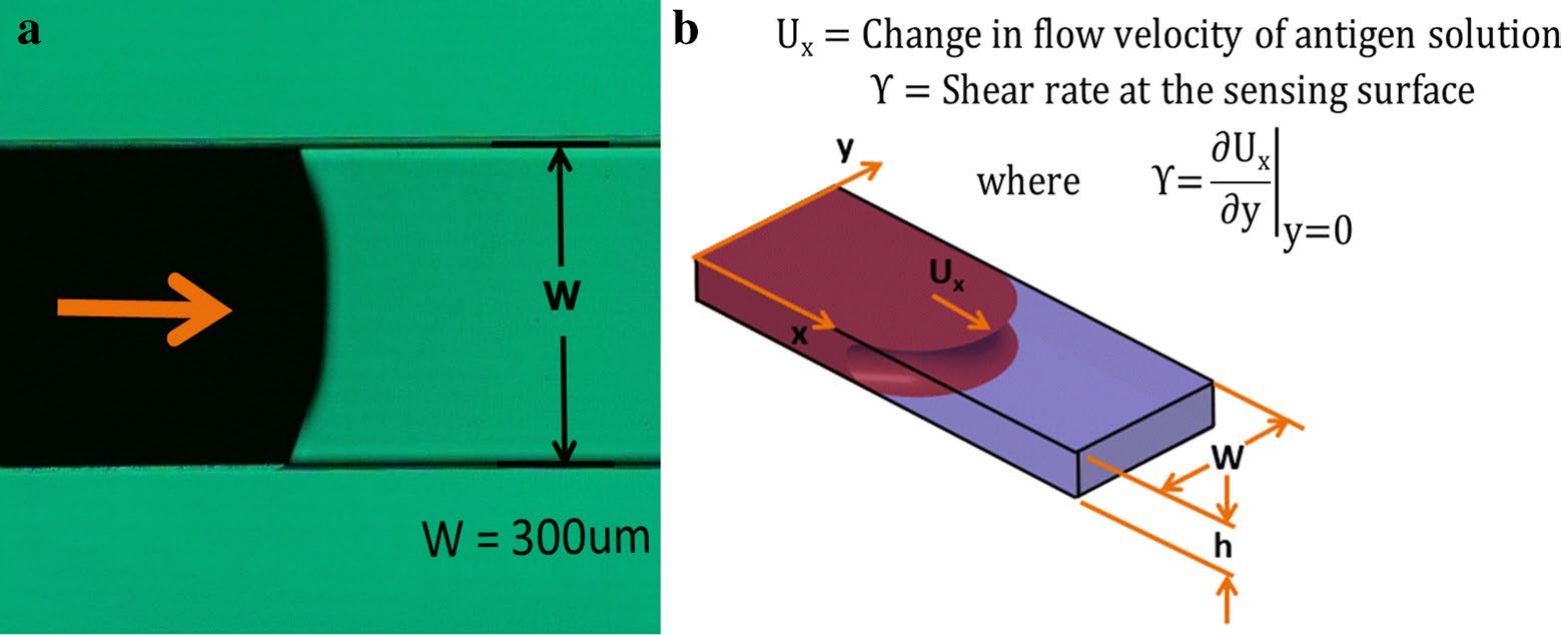
**a** The image microchannel during the flow of antigen solution. **b** Schematic of the microchannel with the shear rate measurement

In this experiment, a microchannel with the width of ‘W’ (300 μm) and depth of ‘h’ (107 μm) is used to generate the microfluidic flow on the sensing platform as shown in Fig. 8a. The microchannel is fabricated using PDMS, which is hydrophobic in nature. The hydrophobic nature of PDMS is converted to hydrophilic using the plasma treatment. The flow of the antigen solution in the microchannel due to the capillary effect generates a shear on the sensing surface. The shear stress at the surface of sensing is defined by the change in the antigen solution flow velocity (U_x_) with respect to the channel height at the channel surface (y = 0), by assuming that the flow of the antigen solution as the poiseuille flow in the infinite parallel plates due to the high aspect ratio and the insignificant side wall effect.

9 $$\tau = \mu \left. {\frac{{\partial {\text{Ux}}}}{{\partial {\text{y}}}}} \right|_{\text{y = 0}} \approx \mu \frac{{ 6 {\text{Q}}}}{{wh^{2} }}$$

where the flow rate of the antigen solution is measured as Q (0.2 μl/s) using a high-speed camera (Phantom V-7.3), with the dynamic viscosity (μ) of the antigen solution (8.8 × 10^−4^ pa s) the width of the microchannel is W (300 μm), the depth of the microchannel is h (107 um) as shown in Fig. 8b. Thus, the shear stress (τ) is calculated as 0.307 pa as per Eq. (9). The shear stress has influences on the stability of the immobilized CA-125 antibodies on top of the sensing surface with gold nano particles and the corresponding effect on sensitivity are explained in detail in Sect. 3.4.5 of the paper.

### Electrical characterization

#### Capacitance measurement of different layers of biosensor

The capacitance is measured at various stages consisting of different sub-layers. All the measurements were taken using a two-point probe station and the dielectric parameters were calculated using Agilent 4284A Precision LCR meter. The frequency range was taken from 10 to 100 kHz for all the sub-layers using 10 kHz steps.

**Fig. 9:**
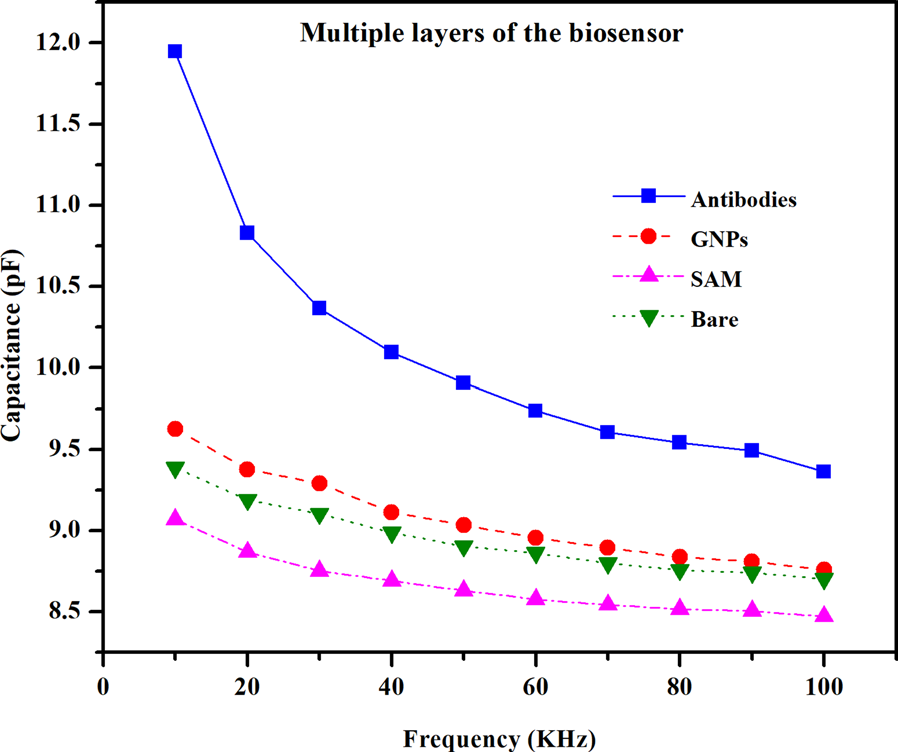
Capacitance variation over frequency for different layers of the sensor

Figure 9 shows the plot of capacitance variation over frequency for different layers of the sensor. The highest capacitance of the bare interdigitated electrodes was 9.38 pF at 10 kHz and the lowest was 8.70 pF at 100 kHz. The capacitance values of the SAM layer (Thiourea) was observed to be lower than the bare electrodes. The highest value recorded was 9.06 pF at 10 kHz and the lowest was 8.47 pF at 100 kHz. The potential reason is the higher charge transfer resistance of the SAM layer which directly affects the real part of the impedance. This increase in the resistance directly influences and increases the net impedance. Because of this phenomenon, the net capacitance of the circuit decreases over frequency [35, 36]. The higher permittivity of the gold nanoparticles resulted in higher capacitance than that of both bare electrodes and the SAM layer, giving values of 9.62 pF at 10 kHz and 8.76 pF at 100 kHz. The capacitance measurement at the immobilized CA-125 antibody layer was 11.94 pF at 10 kHz frequency and then reduced by increasing frequency, down to 9.36 pF at 100 kHz frequency as shown in Fig. 9.

#### Capacitance measurement of a CA-125 antigen conjugation with CA-125 antibodies immobilized on biosensor

The study used a solution of PBS solvent with and without CA-125 antigens respectively. First for the baseline study, plain PBS solution without CA-125 antigens was measured with CA-125 antibodies coated on the nano electrodes. A drop of PBS solution (approximately 1 µl) was placed on the biosensor. The PBS solution capacitance measurements were taken in the frequency range from 10 to 100 kHz. The capacitance values almost remained unchanged over the entire frequency range. The capacitance curve of the plain PBS solution was regarded as the ‘Baseline’. The highest and lowest capacitive values of the ‘Baseline’ curve were measured to be 96.90 pF and 69.19 pF respectively.

**Fig. 10:**
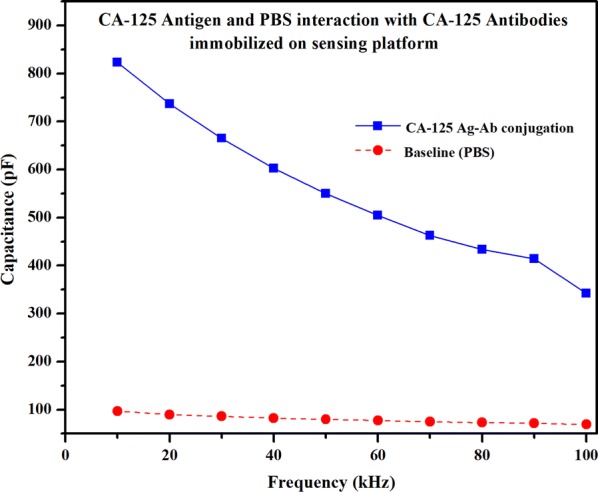
Capacitance variation over frequency for both the cases: the baseline and CA-125 antigens during Ag–Ab conjugation

The next for CA-125 antigens case, a 1 μl droplet of CA-125 antigen solution is placed on the biosensor. The capacitance measurements of the antigen and antibody conjugation are taken from the same range of frequency. The capacitive values of the CA-125 antigen solution case with CA-125 antigen–antibody conjugation, ‘after Ag–Ab conjugation’ curve was measured to be 822.93 pF at 10 kHz and changed to 342.18 pF at 100 kHz. The antigens and the antibodies interaction are very selective and specific. The specific antigen and antibody interactions form a complex which increases the net molecular size. The change in the size of the complex which has a unique property of electrical charge, disturb or interfere the distribution of the charges present in the dielectric medium. The antigen–antibody complex which has unique property of electrical charge creates a change in the distribution of charges within the dielectric region and forms a dipole moment. Because of this phenomenon, the polarization is created due to the dipole–dipole interaction within the dielectric interface [33, 34] substantially leads to a high increase in capacitance. Also, the dielectric values of each antigen–antibody complex over the range of frequencies have its unique variation. The measured impedance or capacitance of the biosensor varies with the change in dielectric properties on sensor surface. The change in the dielectric properties directly influences the change in the capacitance over a range of frequencies. The highest capacitance values were observed at 10 kHz for both ‘Baseline’ and after the Ag–Ab conjugation in the selected frequency range. The capacitance of the ‘Baseline’ is around 96.90 pF and is increased to a value around 822.93 pF after the antigens conjugation at 10 kHz as shown in plot of Fig. 10. The significant change in the capacitance values represents the conjugation of the CA-125 antigens and antibodies.

#### Capacitance measurement comparison of antigen–antibody interactions on plain interdigitated electrodes and gold nanoparticle layered interdigitated electrodes, respectively

**Fig. 11:**
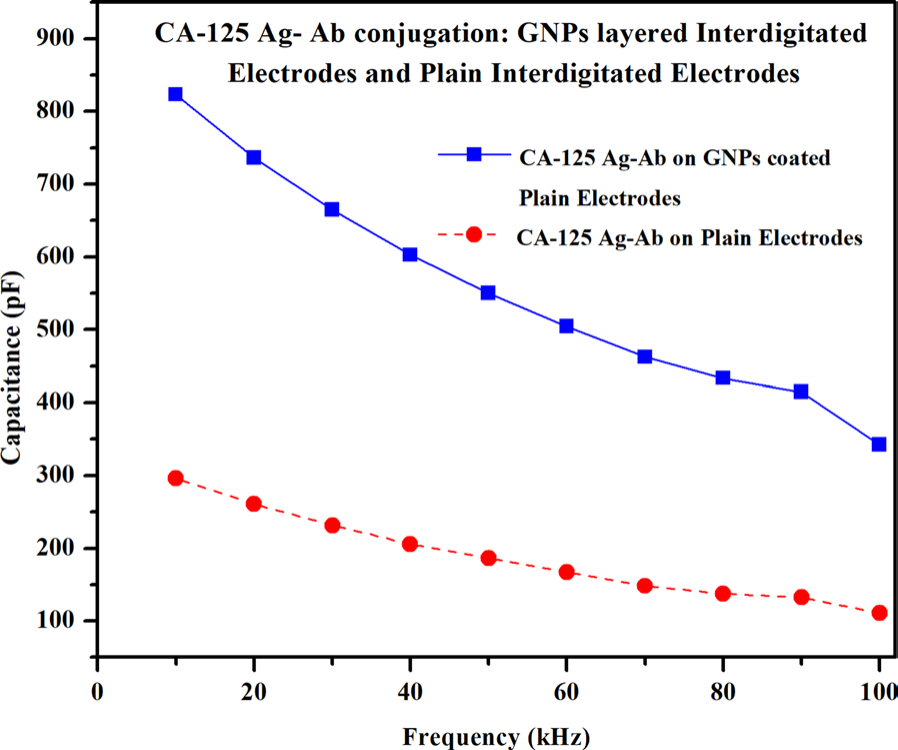
Capacitance variation over frequency for the CA-125 Ag–Ab conjugation on the plain interdigitated electrodes and gold nanoparticle coated interdigitated electrodes

The gold nanoparticles-based sensing platform which was coated with gold nanoparticles on the interdigitated electrodes showed the enhanced capacitance during antigen–antibody interaction, compared to plain interdigitated electrodes, as seen in Fig. 11. Although there are various noble metals which can serve the purpose for biosensing, the gold nanoparticles are found to be promising and better for biosensing due to its unique surface chemistry, high electron densities, chemical inertness and it possesses both good electrical and optical properties [20]. The carboxylic coated gold nanoparticles provide higher surface to volume ratio for the immobilization of the antibodies. The gold nanoparticles provide enhanced stability due to better orientational freedom for the antibodies attachment. This phenomenon not only provides better stability but also results in accumulating more antibodies for the antigen–antibody conjugation. The highest capacitance of both sensing platforms is measured at 10 kHz and are found to be 822.93 pF for gold nanoparticles coated interdigitated electrodes and 296.09 pF for plain interdigitated electrodes. The capacitance of the carboxylic gold nanoparticles is found to be almost three times the capacitance of the plain interdigitated electrodes. Two factors may be contributing to this phenomenon. The first explanation is due to the enhanced orientation freedom and higher surface to volume ratio of the gold nanoparticles as compared to the plain interdigitated electrodes. This characteristic results in increasing the net amount of the antibodies. As a result, the capacitance signal response is higher for gold nanoparticle coated interdigitated electrodes when compared to plain interdigitated electrodes [42–44]. Another explanation is the surface coverage of the carboxylic functionalized group on top of the nanostructure or nano-elements for covalent conjugation. Although the plain interdigitated electrode sensing mechanism also used covalent bonding for antibody binding, the gold nanoparticles provide much more surface area of the electrodes. This resulted in capturing a significantly greater number of antibodies using covalent bonding for the gold nanoparticles-based interdigitated electrodes.

#### Capacitance measurement comparison of antigen–antibody interaction with multiple concentrations of antigens

**Fig. 12:**
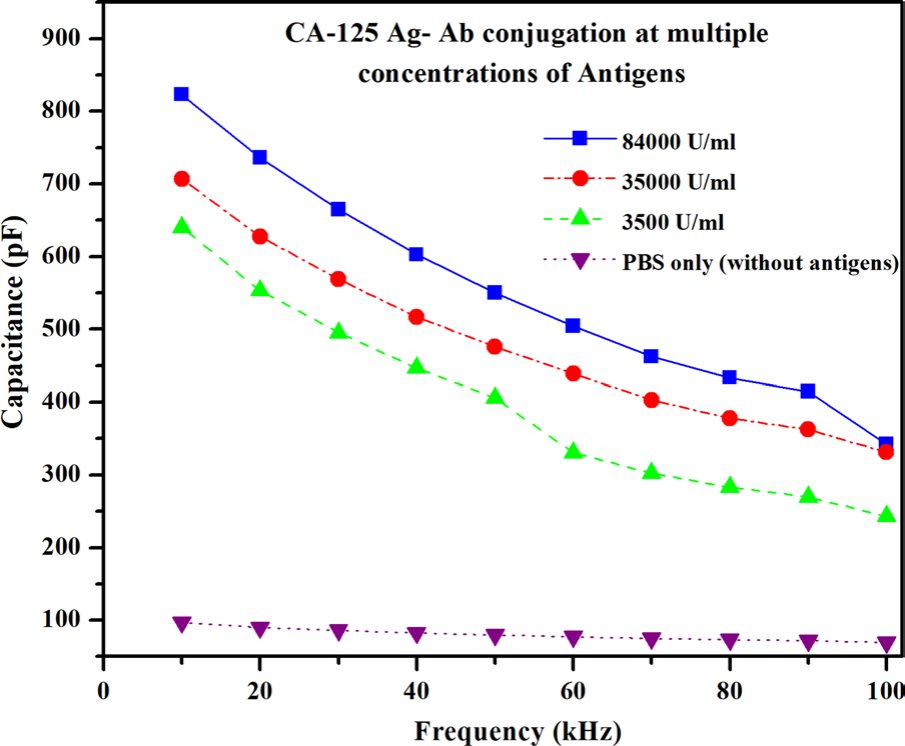
Capacitance variation over frequency for the CA-125 Ag–Ab conjugation with various concentrations of CA-125 antigens and PBS without CA-125 antigens

The capacitance signal response was captured during CA-125 antigen–antibody conjugation with different concentrations of CA-125 antigens, as shown in Fig. 12. The CA-125 antigens are aliquotted into different concentrations: 84,000 U/ml, 35,000 U/ml, 3500 U/ml, and plain PBS (without CA-125 antigens). The CA-125 antigens of different concentrations were measured using the static drop condition on the gold nanoparticle coated interdigitated electrodes. The antibodies immobilized on the gold nanoparticles interact with the CA-125 antigens and generate the corresponding capacitance signal response. The capacitance signal response during the antigen–antibody interaction is as high as 822.93 pF with an antigen solution of 84,000 U/ml and the signal response drops to 706.91 pF, 640.5 pF and 96.90 pF for the concentrations of 35,000 U/ml, 3500 U/ml and PBS (without antigens) respectively at 10 kHz frequency. The capacitance signal response of the PBS solution without any antigens was captured to confirm that the change in signal difference is caused only due to the antigen–antibody interaction. The change in the capacitance signal is directly proportional to the concentration of the antigens in the sample. The signal response of antigen–antibody interaction for lower concentrations of antigens was observed due to the low number of antigens interacting with the antibodies that were immobilized on the gold nanoparticle based interdigitated electrodes. The capacitance signal for all the concentrations decreased as the frequency increased. As predicted by electrochemical theory, the change in the capacitance signal between two different concentrations of antigens at a frequency was almost same (over the frequency range of 10 kHz to 100 kHz).

#### Capacitance measurement comparison of CA-125 antigen–antibody conjugation at static and microfluidic flow condition

The plot in Fig. 12 shows the variation in the signal response between the static condition and the microfluidic flow condition during the CA-125 Ab–Ag conjugation. The carboxylic gold nanoparticles sensing platform without the microchannel (static drop condition) resulted in consistently higher capacitance values because there is no external disturbance on antigen and antibody interaction whereas the microchannel flow has the external effect by the shear of the flow. The highest capacitance is recorded to be 822.93 pF at 10 kHz and the lowest is 342.18 pF at 100 kHz.

##### Microfluidic flow condition of the biofluid sample with CA-125 antigens

**Fig. 13:**
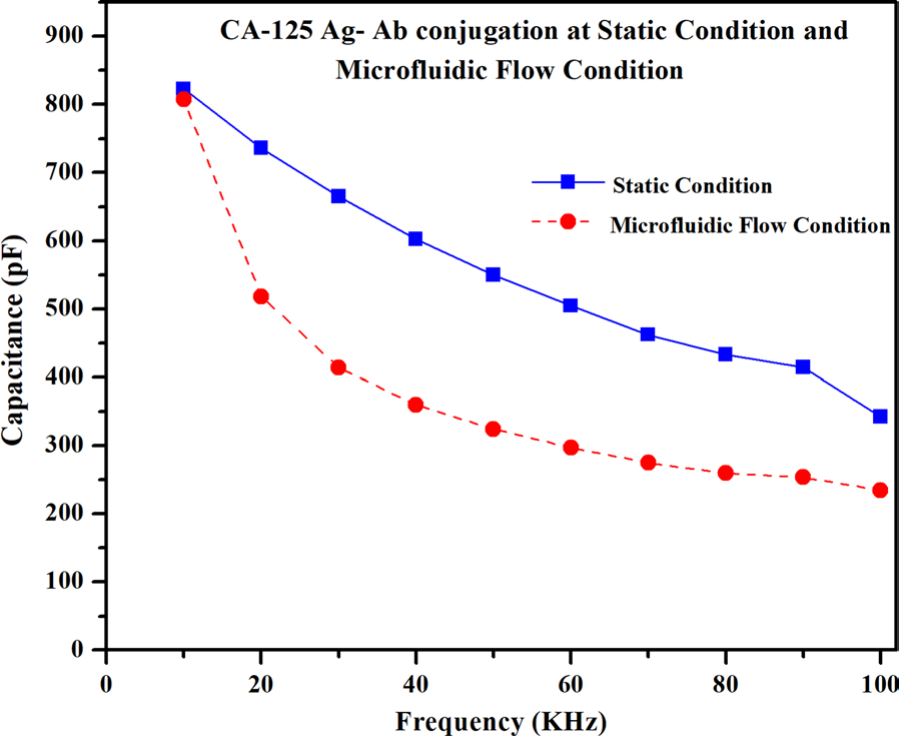
Capacitance variation over frequency for gold nanoparticles under static and microfluidic flow condition

To understand the sensitivity variation due to the microfluidic flow, a biofluid sample with the exact same composition to the static drop condition was passed through the microchannel at a constant flow rate (0.2 μl/s) over the sensing platform. The capacitance values were measured during the antigen–antibody interaction when the biofluid was flowing in the microchannel. The capacitance measurement in the microfluidic flow condition during CA-125 antigen–antibody interaction was measured as 807.30 pF at 10 kHz frequency and gradually decreased to 234.51 pF at 100 kHz frequency as shown in Fig. 13. The capacitance measurement during the CA-125 antigen–antibody interaction has decreased from 822.93 pF for the static drop condition to 342.18 pF at 10 kHz for the microfluidic flow condition as shown in Fig. 13.

The capacitance was recorded from the highest value of 807.30 pF to the lowest value of 234.51 pF within the frequency range from 10 to 100 kHz during the flow of the antigen solution in the microchannel. The tight confinement of the microfluidic flow exerts high surface shear stress which impact the stabilization of the antibodies that are bonded to the sensing platform [37, 38]. The shear forces applied by the fluid on the antibodies that are bonded to the electrode sensing platform in the microchannel induce mechanical breakage of the weak bonds of the antibodies with the electrode [39–41]. The breakage of bonds of the antibodies with the sensing surface could influence the stability of the immobilization of antibodies. So due to the existence of shear in the microfluidic flow condition, the stability of the CA-125 antibody would be significantly lower, which could directly influence the sensitivity. So due to lack of any shear in ‘static’ condition, the stability of the CA-125 antibody was significantly higher and directly enhanced the sensitivity.

##### Capacitance variation during CA-125 antigen antibody interaction at different conditions (at 20 kHz frequency)

**Fig. 14:**
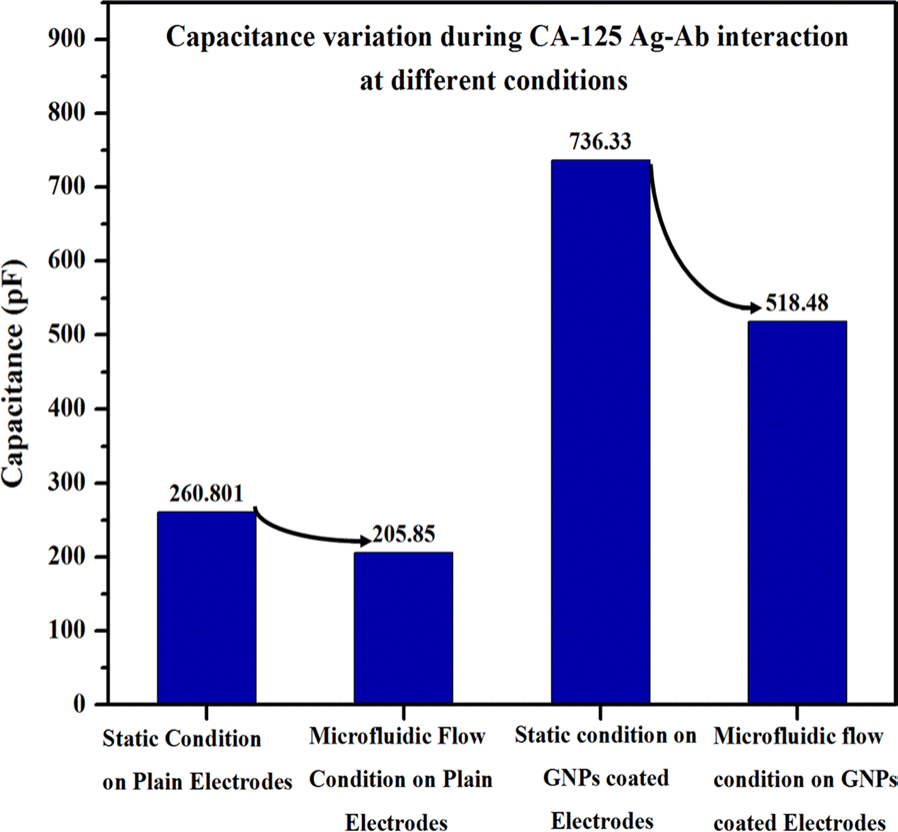
Capacitance variation during CA-125 Ag–Ab interaction at different conditions at 20 kHz frequency

Figure 14 shows the change in the capacitance of antigen–antibody interaction at different conditions of the sensing platform (plain interdigitated electrodes and gold layered interdigitated electrodes) and different flow conditions (static drop condition and microfluidic flow condition) at 20 kHz frequency.

As shown in Fig. 14, the change in the capacitance from the static drop condition to microfluidic condition for plain electrode is 54.95 pF and for gold nanoparticle coated electrodes is 217.85 pF. As explained in the earlier sections, the gold nano particles (GNPs) coated electrodes has the higher sensing signal than the plain electrodes due to the enhanced antibodies immobilization on the gold nano particles with the high surface to volume ratio and orientation freedom. Also, GNPs has the higher resistance to shear flow than plain interdigitated electrodes for microchannel flow.

## Conclusions

The incorporation of microfluidics with biosensing has the following advantages: multiplex assay and simultaneous separation of the targeted biomolecules for detection during the flow, for enhanced signal to noise ratio. However, it has the limitation due to the shear effect caused by the microfluidic flow on the antibodies immobilized on the sensing platform. In the current study, sensitivity variation due to microfluidic flow was established by detecting CA-125 antigens in biofluid using gold interdigitated electrodes. At static drop condition, the signal response of antigen–antibody conjugation in gold nanoparticle-coated interdigitated electrodes is approximately 2.8 times than in plain interdigitated electrodes. At 20 kHz frequency, the signal response of plain interdigitated electrodes during antigen–antibody conjugation has increased from 260.80 to 736.33 pF when the gold nano particles are coated on the plain interdigitated electrodes. At microfluidic flow condition, the signal response of antigen–antibody conjugation in gold nanoparticle-coated interdigitated electrodes is 2.5 times than in plain interdigitated electrodes. At 20 kHz frequency, the signal response of plain interdigitated electrodes during antigen–antibody conjugation has increased from 205.85 to 518.48 pF when the gold nano particles are coated on the plain interdigitated electrodes. Based on the measured results, the following conclusions can be made. (1) The functionality of the individual layers in the sensing platform is validated with the measured change in capacitance. (2) The gold nanoparticle coated interdigitated electrode has higher sensitivity than the plain interdigitated electrode during the CA-125 antigen antibody interaction. (3) The capacitive sensing signal response increased proportionally with the increase in concentration of the antigens during the antigen–antibody conjugation. (4) The effect of shear on the sensing signal response is evident given the lower capacitive signal during antigen–antibody conjugation in the microfluidic flow condition as compared to the static drop condition. The observed effect of shear stress in the microfluidic flow condition during the antigen–antibody conjugation can be mitigated by incorporating the following design changes in the sensing platform and microchannel. (i) The sensing platform with nano well-structure immobilized the antibody into each well, can reduce the shear effect during the microfluidic flow. (ii) The surface treatment to the microchannel for controlling the hydrophilicity of channel reduce the shear caused by the microfluidic flow significantly and thus the effect of the shear on sensing platform can be controlled. Though our focus was on isothermal microfluidic devices, the future work on evaluating the influence of thermal conditions on the sensing signal response in the microfluidic platform would provide additional information regarding the stability of the bioconjugation chemistries in thermocycling microfluidic biosensing applications.

## Abbreviations

CA 125: cancer antigens 125
ml: milliliter
µl: microliter
DNA: deoxyribonucleic acid
kDa: kilodalton
GNPs: gold nano particles
PBS: phosphate buffer saline
PDMS: polydimethylsiloxane
PMMA: poly(methyl methacrylate)
MIBK: methyl isobutyl ketone
IPA: isopropyl alcohol
SAM: self-assembled monolayer
EDC: 1-ethyl-3-(3-dimethylaminopropyl)-carbodiimide
NHS: *N*-hydroxysulfoxuccinimide
mg: microgram
DRIE: deep reactive ion etching
AFM: atomic force microscope
